# Relationship between smoking and health and education spending in Chile

**DOI:** 10.1136/tobaccocontrol-2017-053857

**Published:** 2017-10-06

**Authors:** Guillermo Paraje, Daniel Araya

**Affiliations:** 1 Business School, Universidad Adolfo Ibañez, Santiago, Chile; 2 Universidad Adolfo Ibañez, Santiago, Chile

**Keywords:** disparities, economics, socioeconomic status, health services

## Abstract

**Objective:**

To estimate the degree to which tobacco consumption is associated with spending on a set of goods and services in Chile, especially health and education, for the total population as well as for specific subgroups.

**Methods:**

A seemingly unrelated regression equation system was used to estimate the statistical relationship between having tobacco expenditures and the budget share allocated to other items for the total population and for specific subgroups in Chile (eg, households within the bottom/top 33% by total expenditures). The use of household-level data allows for the control of a number of sociodemographic characteristics. The nationally representative 2012 Chilean Household Expenditure Survey was used for the analysis.

**Results:**

Tobacco consumption is associated with lower budget shares allocated to healthcare, education and housing expenses, especially for poorer households. In the case of health, not consuming tobacco is related to higher health expenditures: up to 32% for the total population. Similarly, in the case of education, not consuming tobacco is statistically related to higher education expenditures: up to 16% for the total population. For all groups, tobacco consumption is also related to a significantly higher budget share allocated to alcoholic beverages.

**Conclusions:**

The strong significant statistical relationship found between tobacco consumption and resources allocated to healthcare and education consumption may be indicative of the existence of a crowding out effect of tobacco. This effect, in turn, may increase the burden that the rest of society must bear for the increased healthcare that they require because of tobacco consumption.

## Introduction

Tobacco consumption is responsible for about 6 million deaths a year worldwide^1^ and some 16 500 deaths in Chile, where it is also directly responsible for the loss of approximately 4 28 000 disability-adjusted life-years per year.^2^ In addition to the harmful impact on health, its consumption has economic consequences at the individual and social level.^3^ On the individual level, it has been noted that there is an apparent link between tobacco consumption and poverty, both present and future: the resources allocated to its purchase are not allocated to the purchase of indispensable goods such as food, clothing or other household expenses; or else they crowd out expenditures on goods and services that have an impact on future well-being, such as education and health, from the family budget.^4 5^ On the social level, the use of tobacco means increased health system expenditures on preventable chronic diseases. In the case of Chile, for example, these costs have been estimated at around 0.8% of GDP.^2^ In addition, indirect costs associated with lost productivity could be added that would significantly increase the above costs.^6^

The case of Chile is particularly interesting due to its status as a high-income, yet developing country (current US$21 300 per capita Purchasing Power Parity (PPP)^7^) with a very high level of tobacco consumption. Chile is the country with the highest monthly smoking prevalence in the region of the Americas, at 35% in 2014.^8^ Monthly prevalence is 36% for men, which is surpassed only by Cuba, while women in Chile have the highest prevalence in the region, with 34%. This prevalence has been on the decline since 2004, when it was 43.6% of the population (46.3% of men and 41% of women). In terms of the socioeconomic distribution, lower-income households had a monthly smoking prevalence of 39.7% in 2014, significantly higher than that of 32.1% in higher socioeconomic levels. This difference between socioeconomic levels has been fairly constant since at least 1998.^8^

There is evidence of tobacco consumption crowding out expenditures on food, education, healthcare and entertainment for middle-income and low-income countries. A study conducted in Cambodia found crowding out effects among urban households in clothing and education (−0.47 and −1.57 percentage points, respectively).^9^ In the case of India, crowding out effects were found on health, travel and durable goods among urban households, in addition to an inverse effect (larger proportion of spending in households that purchase cigarettes) on food, education, fuels, entertainment and alcoholic beverages.^10^ Evidence of crowding out was found in China for food, education, durable goods, agriculture and health, in addition to an inverse effect on spending on transportation and alcoholic beverages.^11 12^ Another study conducted for low-income and middle-income countries found evidence of crowding out in education and health: 8% and 5.5%, respectively.^13^ Similar results have been found for high-income countries.^14 15^

To the best of our knowledge, there are no estimates of this type of effect for Chilean households (or for other countries in Latin America and the Caribbean). The advantage of generating these estimates at the household level lies in the fact that, in addition to identifying the goods that would be affected by spending on tobacco, they allow crowding out to be disaggregated according to sociodemographic variables, such as level of education and sex of head of household, total expenditure level, etc. This allows one to characterise the differential vulnerability of different groups to tobacco consumption.

Like the majority of countries in Latin America, income distribution in Chile is highly concentrated (Gini coefficient of 50.8 in 2011^7^), which means that the households in the lower end of the income distribution receive a very small percentage of total national income. Consequently, these households receive significant subsidies for health, education and social services. For example, the existing public health insurance system in Chile provides lower-income households a full subsidy for healthcare in the public sector, while the rest must contribute a percentage of their income to cover this care. In the case of education, lower-income households tend to attend free primary and secondary education, while the rest of groups contribute partially or totally to the payment of school tuition. Thus, it would be interesting to know what the spending patterns are in said household regarding non-essential expenditures, such as consumption of tobacco or alcohol, especially when consumption of these substances can affect the current and future level of consumption of services that are provided socially.

The objective of this paper is to present the first estimate for Chile of the statistical relationship between positive tobacco consumption and relative spending on a set of goods and services, including food and non-alcoholic beverages, alcoholic beverages, education, health, clothing, housing, transportation, etc. For this purpose, the sociodemographic characteristics of households are considered, in addition to household groups' spending patterns according to their total expenditures. Following international literature, the allocation of the family budget is modelled from a Quadratic Almost Ideal Demand System (QUAIDS). Estimates were made for the general population and population subgroups, using data from the Chilean Household Budget Survey (EPF for its Spanish acronym), carried out in 2011 and 2012, specifically the 33% of households with lowest total expenditure in the population and those belonging to the 33% highest total expenditure in the population.

## The data

The EPF is a survey conducted by the National Statistics Institute every 5 years. Its main objective is to identify the structure and characteristics of consumption among urban households in the regional capitals of Chile and some of its suburban areas with a reference period of 1 year, although the items food, beverages and tobacco have a 2-week recall period.^16^ In the last survey in 2011–2012, the EPF used a sample of 13 056 households, representative at the national level, and provided information on all household spending on almost 1100 goods (revealing, eg, which households consume tobacco and to what extent) and on the socioeconomic and demographic variables of households and all their members. The response rate for daily household expenditures was 83.8%.

Table 1 shows the averages of the major socioeconomic and demographic variables in the EPF, disaggregated by households with and without consumption of tobacco. Among these, households are divided into groups according to whether their spending on tobacco was low (the lowest third of households with a positive budget share for tobacco), medium (the median third of households with a positive budget share for tobacco) and high (the highest third of households in the sample with positive budget share for tobacco).

**Table 1:** Main descriptive variables for the Chilean Household Expenditure Survey 2012

Variables	Total population	No tobacco expenditures	Positive tobacco expenditures	Low tobacco expenditures	Medium tobacco expenditures	High tobacco expnenditures
Percentage of households outside Santiago	42.2% 0.005	44.0% 0.006	38.1% 0.009	41.3% 0.015	39.0% 0.015	33.9% 0.015
Average number of members	3.5 0.017	3.3 0.019	3.9 0.032	4.0 0.053	4.0 0.055	3.7 0.059
Percentage of female household head	40.5% 0.005	41.6% 0.006	37.9% 0.009	35.5% 0.015	40.7% 0.015	37.6% 0.015
Age of household head	52.0 0.151	53.3 0.187	49.2 0.244	47.9 0.404	49.3 0.422	50.3 0.439
Percentage of households with tobacco expenditures	30.7% 0.005	NA NA	NA NA	NA NA	NA NA	NA NA
Average schooling of household head	11.2 0.042	11.2 0.051	11.3 0.073	12.6 0.122	11.2 0.118	9.9 0.125
Average total expenditure (in Chilean pesos)	$8 09 990 8492	$7 68 261 10 016	$9 04 225 15 844	$1 295 993 36 023	$8 83 845 22 478	$5 32 203 12 415
Average budget share in food and beverages (excluding alcoholic beverages)	27.3% 0.002	27.5% 0.002	26.9% 0.003	22.6% 0.004	26.3% 0.004	31.9% 0.005
Average budget share in alcoholic beverages	0.8% 0.000	0.7% 0.000	1.2% 0.000	1.1% 0.001	1.3% 0.001	1.4% 0.001
Average budget share in tobacco	1.1% 0.000	NA NA	3.4% 0.001	0.6% 0.000	2.2% 0.000	7.5% 0.001
Average budget share in clothing	3.6% 0.001	3.3% 0.001	4.2% 0.001	4.9% 0.002	4.3% 0.002	3.4% 0.002
Average budget share in health	5.3% 0.001	5.8% 0.001	4.2% 0.001	5.4% 0.003	4.1% 0.002	3.0% 0.002
Average budget share in education	5.8% 0.001	5.7% 0.001	5.9% 0.002	8.2% 0.003	6.5% 0.003	3.0% 0.002
Average budget share in housing, electricity, heating	17.1% 0.001	18.0% 0.002	15.0% 0.002	13.8% 0.003	15.1% 0.004	16.1% 0.004
Average budget share in transportation	12.4% 0.001	12.1% 0.001	13.1% 0.002	15.0% 0.004	13.5% 0.003	10.8% 0.003
Average budget share in other goods and services	26.7% 0.001	26.9% 0.002	26.1% 0.002	28.5% 0.004	26.9% 0.004	23.0% 0.004

Sample averages, above; SEs, below.

Source: prepared by authors based on EPF.

The table shows that 30.7% of all households register expenditures on tobacco and that on average 3.4% of their total expenditures are on tobacco. Of these households, 38% have women heads of household, less than the average of the households that do not consume. In addition, households that consume tobacco have more members and younger household heads than those which do not. There is no difference in the average schooling (number of years of formal education) between household heads in the two groups, although the average schooling decreases within the group of households that consume tobacco, as the tobacco budget share increases.

In terms of spending, households that consume tobacco have an average total expenditure that is 18% higher than those which do not consume, although the latter allocate a larger proportion of their total expenditures to food, health, housing and other expenses. If t-tests are performed to consider the effect that three levels of smoking intensity have on average household consumption, one can see that some of these differences are statistically significant at more stringent levels. Table 2 displays the results of these tests. A positive mean difference indicates that, for example, households that do not consume tobacco spend a greater proportion of their budgets on food than tobacco consuming households do.

**Table 2:** Mean tests for budget share for households not consuming tobacco minus the budget share of households consuming tobacco‡

	Total households consuming tobacco	Low tobacco consumption	Medium tobacco consumption	High tobacco consumption
Food and beverage (excluding alcoholic beverages)	0.007* 0.003	0.053** 0.005	0.007† 0.005	−0.041** 0.005
Alcoholic beverages	−0.006** 0.000	−0.004** 0.001	−0.006** 0.001	−0.008** 0.001
Clothing	−0.008** 0.001	−0.017** 0.002	−0.009** 0.002	0.001 0.002
Health	0.014** 0.002	0.001 0.003	0.014** 0.003	0.026** 0.003
Education	−0.004* 0.002	−0.026** 0.004	−0.009† 0.003	0.024** 0.003
Housing, electricity, heating	0.029** 0.003	0.041** 0.004	0.025** 0.004	0.019** 0.005
Transportation	−0.009† 0.003	−0.029** 0.004	−0,008* 0.004	0.012* 0.004
Other goods/services	0.011* 0.003	−0.014* 0.005	0.007† 0.005	0.043** 0.005

*p<0.05; **p<0.01; †p<0.1

‡Figure above is the difference of means relative to households with no tobacco expenditures. A positive (negative) figure implies that average budget shares of non-smoking households are higher (lower) than the respective smoking group. Figures are SEs.

These results show that households that do consume tobacco spend a lower proportion of their budget on food, health and items related to the home (in all cases the differences are significant at 95%). On average, households that consume tobacco spend 0.7 percentage points less of their total expenditures on food, a difference that increases for the households with low tobacco consumption levels (5.3 percentage points). Households with a high level of tobacco consumption spend more of their income on food than households that do not consume (4.1 percentage points). Regarding spending on alcoholic beverages, households that consume tobacco allocate 0.6 percentage points more of their expenditures on alcoholic beverage and the higher the level of consumption the greater the proportion allocated to alcoholic beverages.

Given the negative effects smoking has on health, it is particularly important to know the differences in health spending according to household’s pattern of expenditure for tobacco. The results show that households that consume tobacco allocated less of their total spending to health, and the higher the spending on tobacco, the lower the spending on health. The proportion of spending on health that is displaced by tobacco consumption in the high tobacco consumption group is 2.6 percentage points.

Given the structure of the Chilean healthcare system, it is also important to consider the different health insurance systems households can belong to when making this comparison, which can be either public or private. Around 80% of the population is in the public system (Fonasa), while the remainder is in the private system (Isapres), in systems developed for the Armed Forces, or in other smaller systems.^17^ Within the public system, the population is divided into four funds: A, B, C and D. Fund A (which covered 19% of the total population in 2012^18^) corresponds to indigent or low-income individuals who make no payment to the health system; meanwhile, funds B, C and D make payments scaled to their incomes. However, fund B (which accounted for 28% of the total population in 2012^18^) consists of low-income people who receive an almost full subsidy for their healthcare. Thus, household spending on health depends on the health system in which they participate. For groups C and D in the public system and for the private sector, copayments, medicines and medical accessories make up the majority of spending. In the case of groups A and B in the public insurance system, health expenditures are mainly medicines and medical accessories (not covered by public health insurance).

Table 3 classifies households according to the health system to which they belong. As shown in table 2, one can see that households that do not consume tobacco assign a larger sum of resources to healthcare. This is the case in households belonging to all health systems, except for Isapres and the Armed Forces. Within each health system, one can see that in all cases a lower expenditure on tobacco is associated with a higher expenditure on healthcare. In the case of Fonasa groups A and B (those who receive a full subsidy in healthcare in the public sector), one can see that households that consume tobacco spend, on average, 36% and 38% less on health, respectively, compared with households that do not consume tobacco. These differences are even greater in households with high tobacco consumption.

**Table 3:** Total health expenditures (in Chilean pesos) on health by health insurance system and tobacco consumption

	All households	Do not consume tobacco	Consume tobacco	Low consumption	Medium consumption	High consumption
**Total population**						
Health expenditure	$52 829	$52 974	$52 481	$86 924	$48 521	$20 830
Health budget share	5.33%	5.73%	4.36%	5.61%	4.30%	3.12%
**Fonasa group A**						
Health expenditure	$13 875	$13 940	$13 714	$23 357	$12 607	$10 784
Health budget share	3.12%	3.49%	2.22%	2.94%	2.09%	2.04%
**Fonasa group B**						
Health expenditure	$31 511	$33 489	$26 512	$40 852	$27 710	$15 123
Health budget share	5.17%	5.80%	3.58%	4.58%	3.51%	2.93%
**Fonasa group C**						
Health expenditure	$39 599	$44 229	$29 916	$33 179	$36 535	$18 455
Health budget share	5.25%	5.89%	3.90%	3.97%	4.44%	3.16%
**Fonasa group D**						
Health expenditure	$53 263	$54 961	$49 782	$66 152	$56 939	$21 169
Health budget share	6.15%	6.79%	4.85%	6.08%	4.96%	3.23%
**Isapres and Armed Forces**						
Health expenditure	$1 09 349	$1 04 600	$1 20 771	$1 48 955	$1 00 362	$67 448
Health budget share	7.06%	7.11%	6.95%	7.18%	6.40%	7.28%
**Other health systems**						
Health expenditure	$49 844	$51 281	$46 086	$82 696	$46 477	$15 173
Health budget share	4.93%	5.34%	3.86%	5.40%	4.27%	2.24%

Source: prepared by authors based on EPF.

## Methodology

The statistical relationships between households' spending decisions and sociodemographic variables is gauged by estimating Engel curves using QUAIDS, as it has been done by other authors.^9 10 14 19^ This system allows one to simultaneously model the decisions households make and consider whether, controlling for sociodemographic factors, households with positive tobacco expenditures allocate assets differently than those that do not consume tobacco. Furthermore, the specification allows sets of goods to be modelled as luxuries at some expenditure levels and necessities at others. Ceteris paribus, positive tobacco expenditures mean fewer resources available for other goods. A less trivial matter is how remaining resources are relatively allocated among different sets of goods and whether this relative pattern differs among households that consume tobacco consumption and those which do not. Thus, budget shares for different categories are considered by dividing what households spend on these categories by the total household expenditures net of tobacco.^10 14^

Thus, a household’s decision on the proportion of spending allocated to different goods is:

$$w_{ih}=\alpha +\beta *Tobacco_{h}+\gamma *Demo_{h}+\varepsilon_{ih}$$

where w_ih_ is the proportion of total household expenditure (net of tobacco) allocated to good *i* by household *h*, in which *i* can be food and non-alcoholic beverages, alcoholic beverages, clothing, health, education, housing and transportation; *Tobacco* is a dichotomous variable that takes the value 0 if the household does not spend on tobacco and one otherwise; *Demo* is a vector of sociodemographic variables at the household level, including the logarithm of total household expenditure, the logarithm of total expenditure squared (to consider non-linear relationships between total expenditure and allocation of goods), the logarithm of the number of people in the household; the sex, age and the age squared of the head of the household, and his or her level of education. The last of these variables is considered with a set of dichotomous variables that measure whether the head of household has incomplete secondary; complete secondary, or some form of tertiary/university education (complete or incomplete). These covariates are extensively used in similar articles to control for socioeconomic differences, with some variation in the total household expenditure variable. Some authors consider total household expenditure,^9 19^ as we do, while others use the total household expenditures net of tobacco.^14 20^

Although some authors claim that β, as estimated here, measures crowding out of expenditures, implying a causal relationship between positive tobacco expenditures and a differential budget share allocation,^9^ others claim that this parameter may be endogenous and that instrumental variables must be used to estimate such a crowding out.^10 14 19^ Unfortunately, the instrumental variables they propose are not available in the EPF and therefore, endogeneity cannot be ruled out. Thus, one cannot accurately state that that β measures crowding out here, but rather the differential proportion of the total net household expenditure that households with positive tobacco expenditures allocate to a certain category vis-á-vis households with no tobacco expenditures. In other words, no causality is implied in the estimated model.

Given that households simultaneously decide the proportion of their income spent on various goods and are constrained by a single household budget, spending decisions in one category affect spending decisions in the remaining categories. Therefore, errors in *ε_ih_* are correlated with each group of goods and the expenditure equations must be estimated simultaneously. For this reason, they are estimated using Seemingly Unrelated Regression Equations (SURE), which accounts for this correlation between the various equation errors.^9 20^ The other set of expenses not considered are eliminated from the SURE estimate to ensure that the conditions underlying household budget constraints are fulfilled.^9^

The SURE is estimated for the total number of households in the sample; for the lowest 33% of households according to total expenditure and for households whose total expenditures are among the 33% highest. This is done to account for significant dispersion in terms of the expenditures that these households might display in a country with a high concentration of incomes/expenditures (the Gini coefficient for total household expenditures is 0.48 and for per capita total household expenditure is 0.50).

The choice of these groups is discretionary, as there is no objective rule for doing so. The main results obtained are robust to different groupings, such as the one used by the ‘Palma ratio’ (bottom 40% and top 10% of the expenditure distribution).^21^

## Results

The results of the SURE models can be found in table 4. The top of the table shows the results for the total population, the middle pane contains the results of the households in the first tertile (33% lowest total expenditure) and the bottom pane has the results of the households in the top tertile (33% highest total expenditure). In all cases, the result of *β* stands out: a positive (negative) coefficient is associated with a higher (lower) budget share in that group of goods on the part of households that consume tobacco. The results obtained do not imply causality (ie, positive smoking expenditures cause differential budget allocation), but they do reveal statistical associations.

**Table 4:** SURE estimates for budget shares for total population and bottom/top 33% of population

Variables	(1)	(2)	(3)	(4)	(5)	(6)	(7)
	Share food and beverages (excluding alcohol)	Share alcoholic beverages	Share clothing	Share health	Share education	Share housing	Share transportation, etc
**Total population**							
Positive tobacco expenditures (ref: no tobacco expenditure)	0.0241** (0.003)	0.0067** (0.000)	0.0050** (0.001)	−0.0135** (0.002)	−0.0092** (0.002)	−0.0068** (0.003)	**−0.0001** (0.002)
Log total household expenditure	−0.2175** (0.028)	0.0146** (0.004)	0.0712** (0.014)	0.1147** (0.018)	0.0600** (0.021)	−0.0582* (0.027)	−0.1575** (0.025)
Log total household expenditure squared	0.0046** (0.001)	−0.0005** (0.000)	−0.0021** (0.001)	−0.0035** (0.001)	−0.0017* (0.001)	0.0003 (0.001)	0.0078** (0.001)
Log number of household members	0.0556** (0.003)	−0.0033** (0.000)	−0.0015 (0.001)	−0.0156** (0.002)	0.0351** (0.002)	−0.0212** (0.003)	−0.0133** (0.002)
Sex of household head (ref: male)	−0.0148** (0.003)	−0.0029** (0.000)	0.0034** (0.001)	0.0099** (0.002)	0.0123** (0.002)	0.0095** (0.002)	−0.0217** (0.002)
Age of household head	0.0028** (0.000)	0.0001 (0.000)	−0.0003 (0.000)	−0.0004 (0.000)	0.0015** (0.000)	−0.0030** (0.000)	0.0008† (0.000)
Age of household head squared	−0.0000** (0.000)	−0.0000 (0.000)	−0.0000 (0.000)	0.0000** (0.000)	−0.0000** (0.000)	0.0000** (0.000)	−0.0000** (0.000)
Education of household head: secondary incomplete (ref: some tertiary/university education)	0.0688** (0.004)	0.0003 (0.001)	0.0116** (0.002)	−0.0079** (0.003)	−0.0371** (0.003)	−0.0248** (0.004)	0.0224** (0.004)
Education of household head: secondary complete (ref: some tertiary/university education)	0.0280** [0.004]	−0.0002 [0.001]	0.0095** [0.002]	−0.0030 [0.002]	−0.0216** [0.003]	−0.0228** [0.004]	0.0283** [0.003]
Observations	10 490	10 490	10 490	10 490	10 490	10 490	10 490
R^2^	0.400	0.033	0.053	0.093	0.111	0.142	0.134
**Bottom 33% of population expenditure**							
Positive tobacco expenditures (ref: no tobacco expenditure)	0.0300** (0.006)	0.0084** (0.001)	**0.0007** (0.002)	−0.0166** (0.002)	−0.0138** (0.003)	−0.0132* (0.006)	**0.0055** (0.004)
Log total household expenditure	0.0336 (0.119)	−0.0269 (0.016)	−0.1689** (0.042)	−0.0343 (0.043)	−0.0990 (0.061)	0.1095 (0.102)	0.1261† (0.069)
Total household expenditure squared	−0.0052 (0.005)	0.0011 (0.001)	0.0077** (0.002)	0.0021 (0.002)	0.0048† (0.003)	−0.0065 (0.004)	−0.0041 (0.003)
Log number of household members	0.0440** (0.008)	−0.0018 (0.001)	−0.0053† (0.003)	−0.0081** (0.003)	0.0184** (0.004)	−0.0065 (0.007)	−0.0049 (0.005)
Sex of household head (ref: male)	−0.0132* (0.006)	−0.0019* (0.001)	−0.0014 (0.002)	0.0036† (0.002)	0.0036 (0.003)	0.0109* (0.005)	−0.0085* (0.003)
Age of household head	0.0015 (0.001)	0.0000 (0.000)	−0.0004 (0.000)	−0.0002 (0.000)	0.0033** (0.001)	−0.0014 (0.001)	0.0004 (0.001)
Age of household head squared	−0.0000 (0.000)	−0.0000 (0.000)	0.0000 (0.000)	0.0000 (0.000)	−0.0000** (0.000)	0.0000† (0.000)	−0.0000 (0.000)
Education of household head: secondary incomplete (ref: some tertiary/university education)	0.1058** (0.013)	−0.0022 (0.002)	0.0056 (0.005)	−0.0148** (0.005)	−0.0392** (0.007)	−0.0132 (0.011)	−0.0038 (0.008)
Education of household head: secondary complete (ref: some tertiary/university education)	0.0622** (0.013)	−0.0025 (0.002)	0.0044 (0.005)	−0.0110* (0.005)	−0.0252** (0.007)	−0.0081 (0.011)	0.0035 (0.008)
Observations	3241	3241	3241	3241	3241	3241	3241
R^2^	0.113	0.030	0.044	0.054	0.086	0.073	0.047
**Top 33% of population expenditure**							
Positive tobacco expenditures (ref: no tobacco expenditure)	0.0219** (0.003)	0.0053** (0.001)	0.0069** (0.002)	−0.0139** (0.003)	**−0.0026** (0.004)	**−0.0010** (0.004)	**−0.0026** (0.005)
Log total household expenditure	−0.5597** (0.069)	0.0038 (0.013)	0.1382** (0.050)	0.3127** (0.076)	−0.2898** (0.081)	−0.2441** (0.086)	0.3360** (0.109)
Total household expenditure squared	0.0170** (0.002)	−0.0002 (0.000)	−0.0047** (0.002)	−0.0107** (0.003)	0.0097** (0.003)	0.0074* (0.003)	−0.0093* (0.004)
Log number of household members	0.0522** (0.004)	−0.0022** (0.001)	0.0015 (0.003)	−0.0082† (0.004)	0.0830** (0.004)	−0.0404** (0.005)	−0.0358** (0.006)
Sex of household head (ref: male)	−0.0169** (0.003)	−0.0033** (0.001)	0.0074** (0.002)	0.0163** (0.003)	0.0243** (0.004)	0.0077* (0.004)	−0.0343** (0.005)
Age of household head	0.0041** (0.001)	0.0001 (0.000)	0.0005 (0.000)	−0.0009 (0.001)	−0.0036** (0.001)	−0.0048** (0.001)	0.0030** (0.001)
Age of household head squared	−0.0000** (0.000)	−0.0000 (0.000)	−0.0000* (0.000)	0.0000** (0.000)	0.0000** (0.000)	0.0000** (0.000)	−0.0000** (0.000)
Education of household head: secondary incomplete (ref: some tertiary/university education)	0.0461** (0.005)	0.0003 (0.001)	0.0146**(0.003)	0.0007 (0.005)	−0.0415** (0.006)	−0.0196** (0.006)	0.0369** (0.008)
Education of household head: secondary complete (ref: some tertiary/university education)	0.0197** (0.004)	−0.0005 (0.001)	0.0097** (0.003)	−0.0035 (0.004)	−0.0303** (0.004)	−0.0225** (0.005)	0.0426** (0.006)
Observations	3747	3747	3747	3747	3747	3747	3747
R^2^	0.304	0.041	0.025	0.091	0.153	0.161	0.108

*p<0.05; **p<0.01; †p<0.1.

Bold values are the B (beta) coefficients of the estimated model.

SEs in paranthesis.

For the total population and the group with lowest total expenditures alike, the results show that households that spend on tobacco allocate a smaller share of their budgets to healthcare, education and housing expenses. The results are statistically significant in all cases (at least for p<0.05). In the case of healthcare expenditures, one can see that at the level of the total population, households that consume tobacco allocate 1.35 percentage points less of their budget (net of tobacco expenditures) to health. Considering that they allocate 4.2% of their budgets to such expenses (see table 1), one could say that if they did not spend on tobacco they could, for instance, increase their health expenditures by 32%. In the case of households in lower tertile (bottom 33% of households), this percentage would be even higher.

In the case of education expenditures, households with tobacco consumption allocate 1 percentage point less of their net budget than non-smoking households, for the whole of the population, and 1.4 percentage point in households in the lowest tertile. Considering that households that consume tobacco allocate 5.9% of their budget to education, this percentage point would imply, ceteris paribus, that they could increase their education budgets by 16% if they did not spend on tobacco.

Both healthcare as well as education expenditures have a heavily-associated ‘total expenditure effect’, although it declines with the level of total expenditure for the general population. As can be seen in the table, the coefficient of the logarithm of total expenditure is positive and significant, which means that, controlling for the other variables, as total household expenditure increases, the budget share allocated to education and health increases, although non-linearly.

Lastly, for both the total population as well as the two population groups considered, households that consume tobacco allocate a larger budget share to expenditures on alcoholic beverages. At the population level, this means an additional 0.7 percentage points allocated to the budget for alcohol. Considering these households' expenditures, this would imply doubling what non-smoking households allocate to alcohol out of their net budget.

## Discussion

The results obtained for Chile are in line with those obtained in similar studies for other low-income and middle-income countries, although Chile’s average income level is substantially higher than the countries studied in other cases,^9 11–13 20^ and also for high-income countries.^14 15^ Findings also suggest that in Chile relative expenditures on healthcare and education, among other things, are significantly lower for smoking households, after controlling for a number of socioeconomic variables. Although the model estimated does not imply causality (ie, that smoking causes lower expenditures on healthcare and education), the statistical associations found have at least two public policy implications. The first is related to the current level of household well-being:smoking households spend less on healthcare (for whatever reason), both preventive and curative, which may worsen the health of smokers and their families, increasing the weight that the rest of society must bear for the increased healthcare that they require. This is made even worse when one considers that households with positive tobacco expenditures also are the biggest spenders on alcoholic beverages, which could amplify the negative consequences on their health and family budget.^11 22^

The second implication deals with future individual and social well-being. Since a significant portion of society receives government-funded subsidies to increase their stock of human capital in the form of healthcare and education provision, expenditures on substances that reduce the stock of human-social capital ultimately have consequences for society as a whole in addition to individuals. Thus, implementing measures to reduce the consumption of such substances (eg, taxes) could have a positive effect on individual and social human capital attainment and would eventually have a positive impact on economic growth.^23^

Starting in 2006, when Chile ratified the Framework Convention on Tobacco Control (FCTC), a series of measures were implemented to reduce tobacco consumption.^24^ Crucially, the real price of tobacco increased sharply: 66% in real terms between early 2006 and the end of 2012 and 114% between early 2006 and the end of 2015. These measures seem to have had an effect, as smoking prevalence was reduced, although Chile remains among the highest-consuming countries in the Americas. Lowering the prevalence of tobacco consumption even further must entail deploying more aggressive control measures, including increased tobacco taxes. If tobacco consumption is responsible for the differential budget allocation, these taxes could save lower-income households significant resources,^25^ and contribute to boosting the country’s economic growth and development by increasing human capital while reducing lost productivity due to health problems. With these interventions, therefore, the government could reduce future healthcare expenses while increasing revenues, households would allocate greater budget shares to healthcare and education while reducing alcohol consumption, and Chilean society as a whole would benefit from a more rapid accumulation of human capital.

**What this paper adds**

- Tobacco consumption is related to crowding out of different items in several low-income and middle-income countries (China, India, Cambodia, etc), but to the best of our knowledge there are no estimates of such effects for Chile or any other Latin American country. Despite the high prevalence of smoking in Chile, it is not known how smoking is related to different socioeconomic-related consumption patterns.
- Tobacco consumption in Chile has a strong, statistically significant relationship to lower budget shares allocated to health and education and this effect is stronger in poorer households. Tobacco consumption is also related to higher budget share allocated to alcoholic beverages. This could amplify the burden that tobacco consumption places on social health expenditures.
